# New products

**DOI:** 10.1155/S1463924688000288

**Published:** 1988


Journal of Automatic Chemistry, Vol. 10, No. 3 (July–September 1988), pp. 147–156

### New products

### IDAS

A versatile real-time data acquisition system has been added to Beckman's 'CALS' laboratory automation system.

The IDAS (Instrument Data Acquisition System) provides two-way communication between virtually any analytical instrument and a DEC VAX, HP1000 or IBM S/370 host minicomputer.

IDAS is programmed to meet the requirements of a variety of instruments by using LIL – a specially developed laboratory interface language. IDAS procedures can be written for prompting the instrument for data and also collect, parse, format and write the data to disk. If the CALS Lab Manager LIMS is running on minicomputer, IDAS is capable of posting data to the Lab Manager data-base. Consequently, significant productivity improvements can be expected from the connection of analytical instrumentation and robotic work cells directly to the Lab Manager data-base.

IDAS can be employed over a direct RS-232 link between an instrument with an on-board programmable computer and an asynchronous port on the minicomputer. Similar connections can be made to instruments with IBM PC workstations with an unused RS-232 communications port – and to various Beckman instruments such as LS 5801 Series liquid scintillation counters and DU-60 Series spectrophotometers.

IDAS can also be installed using the Beckman MK5S Digimetry programmable coupler. This device includes an on-board microprocessor, memory, keyboard, LCD and eight RS-232 input/output ports.

*Details from Beckman, Progress Road, Sands Industrial Estate, High Wycombe, Buckinghamshire, UK. Tel.: 0494 41181.*

### VYDAC HPLC columns

HPLC Technology Ltd (UK) announce the availability of the complete range of Vydac HPLC columns (manufactured by the Separations Group, USA). Not only is the Vydac range now available – but at prices that are 20% lower than previously seen in the UK.

The Vydac columns have almost become an industry standard for protein and peptide analysis and the ion chromatography columns have been extensively used for cation and anion analysis.

*Price list from HPLC Technology Ltd, Wellington House, Waterloo Street West, Macclesfield, Cheshire SK11 6PJ, UK. Tel.: 0625 613848; fax: 0625 616916.*

### Technical curve fitting and plotting software

Aston Scientific's Techni-Curve now offers a number of new features. These include a high-quality print output option on Epson or IBM dot matrix printers, disk storage of plot files and a quantitative analysis routine allowing unknowns to be evaluated from fitted curves.

All existing users can benefit from these enhancements at no charge.

The price of Techni-Curve remains unchanged at £195.00 and a 'live' evaluation package is available at £25.00.

*More information from Martin Perry, Aston Scientific Ltd, 49 Long Plough, Aston Clinton, Buckinghamshire HP22 5HD, UK. Tel.: 0296 630304.*

### Alphanumeric recorders

H-S Recorders have announced two additions to their range of Flexicorder ulta-lightweight alphanumeric waveform recorders. The use of a new micro stepping motor drive circuit gives the flexibility to provide a wider variation of chart speeds and reduces both mechanical and electrical noise at all speeds.

This IBM compatible module is designed specifically for control and monitoring functions in medical, scientific, and industrial equipment, and the recorders can provide alphanumeric text, single or dual waveform information, and graphics. In addition, they also have the ability to overlap text or annotations across the waveforms so that they relate directly to particular points or events.

The 50 mm-wide model - coded HS50MC – uses a full width solid state thermal print head giving 288 addressable dots across 48 mm of a standard 50 mm wide paper roll. The 63 mm version (HS60MC) gives 320 dots across 53 mm of a standard 63 mm wide paper roll. Both units are powered by a compact stepper motor micro-stepped drive to provide a high torque/low noise combination, whilst allowing the selection of varying motor speeds.

Both recorders can operate at any one of eight standard speeds ranging from 1 mm/min to 50 mm/s, or infinitely variable speed control between 0 and 25 mm/s is possible by use of an external clock.

Waveform information can be read at the rate of 5000 samples/s or smoothed at rates down to 20 samples/s by the use of host selectable interpolation. Text printing on both units can use any of three character sizes vertically and four character sizes horizontally. In addition, both have a high resolution graphics facility.

Resolution is enhanced by a density of 24 dots/mm along the paper roll. Software selectable trace expansion allows traces to be multiplied by 1·25

and 1·125 for the HS60MC and the HS50MC respectively and this gives each recorder a full grid width trace.

Other features of both units include built-in self test for thermal, mechanical, and anti-static print head protection; IBM compatibility and recording control via software or specially developed interfaces.

*Full details of the new specification HS50MC and HS60MC recorders can be obtained from H-S Recorders Ltd, Unit 51, Portmanmoor Road Industrial Estate, Cardiff CF2 2HB, UK. Tel.: 0222 485885; fax: 0222 462173.*

### Colour measurement

The Vector's CL6000 colour measurement system is being promoted as the best buy low budget system. A four element filter set mounted in a mechanically and thermally stable housing, enable reproducibility and stability of results to be better than 1%.

Reflectance and transmittance heads for solids or liquids are available, the transmittance head is designed for cuvettes up to 50 mm.

The 80186 based control system is packaged in a housing suitable for laboratory or production environments.

Results can be sent to an external computer, including PC/AT/XT systems, for which software is available.

Level 4, the top of the range, is a fully integrated colour management system for total factory control and reporting.

*Details from Trivector Systems International Ltd, Sunderland Road, Sandy, Bedfordshire SG19 1RB, UK. Tel.: 0767 82222.*

### Automation with PCs

PCs, of course, are being used by many organizations for low-cost automation, by adding the right interface hardware and software. However, before he can exploit this opportunity, the scientist or engineer must first research the market and find the most suitable products. He may resort to writing his own software to make the system work and this can end up taking more time than the system will save.

Industrial Data Processing Ltd have introduced a service to help scientists and engineers benefit from this technology without wasting time. IDP are experts in the use of the PC for automation, and offer a complete service including advice as to how best to apply the technology, which equipment to use and the most suitable software. The company also supplies systems 'off the shelf' and takes complete responsibility for providing time-saving solutions, including installation, commissioning and training.

I.D.P. base their solutions on standard IBM PCs or compatibles and interface hardware from Burr Brown, Analog Devices, Metrabyte, National Instruments or Techmar. The company run monthly seminars to demonstrate how these systems can be used.

*More information from Kevin Hickey, Industrial Data Processing Ltd, The Maltings, High Street, Burwell, Cambridge CB5 0HB, UK. Tel.: 0638 743044; fax: 0638 743066.*

### Automatic vision inspection system

The Trivector Comparatronic 5124 provides reliable on-line inspection of products at rates up to 1500/min. The system is based on a framestore with a resolution of 512 × 512 pixels by 4 bits (16 grey levels). The Comparatronic 5124 operates by direct subtraction of the real-time image of an object from a stored master image. If the two images are identical then they cancel each other out completely and the product is accepted. If the two images differ, then the difference will be measured, against preset thresholds of tone value difference and defect size, and if the difference exceeds these thresholds, the product will be rejected.

The versatility of the Comparatronic 5124 system can be expanded by the addition of a Compu-Scan Image Handling System. This consists of a computer, a framestore and disk storage, and provides several quality-control functions. Linked to a Comparatronic 5124, it can acquire the image of any defective product and apply a series of image processing algorithms to that image to determine the nature and seriousness of the defect. A report can be generated, corrective action triggered and the image can be either stored or transmitted down a telephone line. Typical applications for the Comparatronic 5124 include label and packing checking.

*More information from Trivector Systems International Ltd, Sunderland Road, Sandy, Bedfordshire SG19 1RB, UK. Tel.: 0767 82222.*

### Kjeldahl-Automat

An automatic Kjeldahl system from BüCHI provides automatic distillation, titration, calculation and printout of the results including full operational parameters. The unit offers the benefits of high throughput with excellent accuracy and reproducibility.

The B-343/322 is extremely easy to use. Using a dialogue-driven menu (available in English, German and French) one of four operational methods may be chosen. The results can be printed in up to seven ways.

A balance, printer and computer can be plugged into the B-343 using standard connections.

*Details from BüCHI Laboratory-Techniques Ltd, PO Box, CH-9230 Flawil, Switzerland. Tel.: 071 84 81 81; fax: 071 83 59 11.*

### Determination of pK-values

The VIA100 pK method module for installation in the TitraLab high-performance titration system. With the pK Method Module installed, the TitraLab system is capable of calculating pKa-values from the data obtained during a titration.

The pKa-value is calculated on three measuring points around the half neutralization point. The mean value is listed after the titration. In addition to the pK-value the corresponding titration result calculated is listed.

Up to four pK-values with related concentration can be determined in one titration.

The pK method module is especially useful in research laboratories for pKa determinations on newly developed species, and in quality control laboratories where the pK-value listed with the titration result ensures that the correct compound has been titrated.

*Details from Henrik Malmvig, Radiometer Copenhagen, 49 Krogshøjvej, DK 2880 Bagsvaerd, Denmark. Tel.: 01 69 63 11; fax: 02 49 00 11.*

### Software

KNOWLEDGEMAN is a data-management system from Jobin Yvon. The software was written by Micro Data Base Systems, and was then tailored by Jobin Yvon to provide data management, production of reports with text and graphics, and also to perform statistical calculations on data acquired with a JY ICP spectrometer. It is an open software, which can be customized.

Level 1 contains KMAN with relational data management, spur-of-the-moment enquiry, spreadsheet facility, statistical analysis programmes and a built-in calculator; KGRAPH with 15 styles of graphs available, and KTEXT, a word-processing package. Level 2 is the customized version where JY software engineers write additional programs on request. The package operates under MS-DOS on the IBM-PS2 (or compatible) computers.

Level 1 is supplied as standard with the Plus versions of the ICP range but both levels are available as options with any of the sequential, simultaneous and combined ICP systems.

*For more information contact Dr Maryanne Thomsen, Instruments SA - EDT Ltd, 14 Trading Estate Road, London NW10 7LU. Tel.: 01 965 8500; fax 01 961 9210.*

### Apex PrepSil

Apex PrepSil, from Jones Chromatography, is a high-quality HPLC material for preparative chromatography, available in 8 μ, 15 μ and 20 μ sizes, in C8, C18 and plain silica. It is analogous to the 3 μ, 5 μ and 10 μ Apex materials, allowing easy scale-up from analytical to preparative chromatography.

Apex PrepSil materials are available as bulk materials and in packed columns.

*For further information contact Jones Chromatography Ltd, New Road, Hengoed, Mid Glamorgan CF8 8AU, UK. Tel.: 0443 816991; fax 0443 816552.*

### Micropump drive catalogue

The 5003U Micropump drive, and the compatibility of Micropumps with a broad range of chemicals and solvents, are described in a catalogue from Watson-Marlow.

Sharing many of the advantages of peristaltic pumps including self-priming, dry running, non-contaminating and a positive displacement action, Micropumps are the preferred pump type at high pressures (up to 20 bar and differential pressures of 8 bar) and flow rates (up to 4600 ml/min). In addition, Micropumps may be used for liquid/air mixtures, solvents which could corrode plastic tubes, provide a virtually pulseless flow, operate at high temperatures and are leak proof.

Micropumps to fit the Watson-Marlow 5003U drive are listed, and plots of flow rates against pressure are included.

The Micropump body, seal and gears are available in a range of materials to cope with any solvent or chemical.

*Details from Smith and Nephew Watson-Marlow, Falmouth, Cornwall TR11 4RU, UK. Tel.: 0326 73461; fax: 0326 76009.*

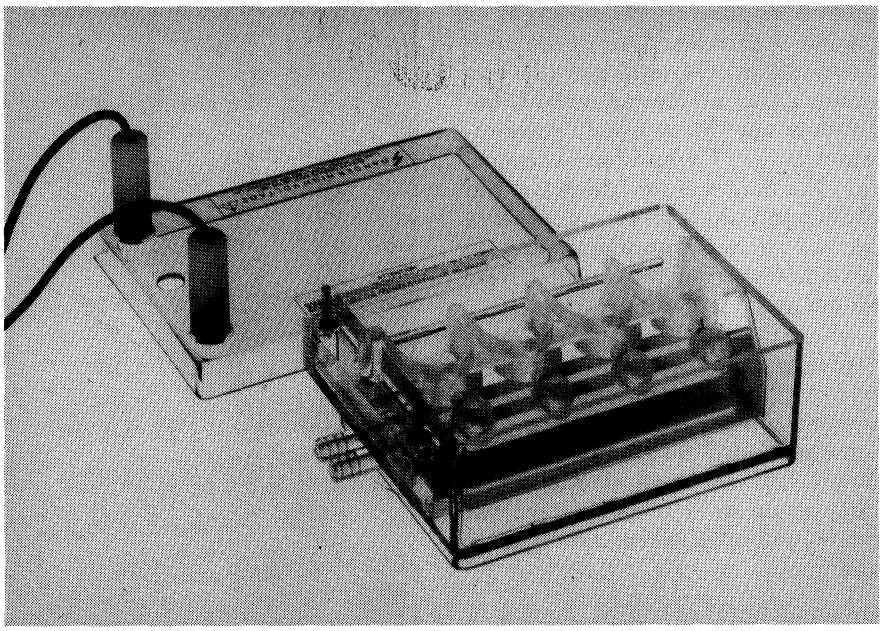

*Biometra's Elucon. Advantages of this electrophoretic elution and concentration equipment for proteins and other biological macromolecules present in gels include:*

- *Easy handling and many applications (electron-elution; electro-concentration; electro-ultrafiltration).*
- *Concentrations in small volumes (20–10 μl).*
- *Four quick elutions in parallel.*
- *Sample cooling.*
- *Small membrane surface.*

*Details from Christel Wallman, Biometra Biomedizinsche Analytik GmbH, Wagenstieg 5, 3400 Göttingen, FR Germany. Tel.: 0551 37 10 32 34; fax: 47 655.*

### Optical emission technique

ARL has recently issued a poster which describes the technique used by atomic emission spectrometers. The chart illustrates the various excitation techniques, polychromator and monochromator, measuring electronics and analysis procedure. This very useful guide to the technique of OE, in English, French and German, has also been produced on a format small enough for handing-out.

*Copies from ARL – Applied Research Laboratories SA, En Vallaire, 1024 Ecublens, Switzerland. Tel.: 021 34 97 01.*

### Photophone – Picture telephone

Ciba-Geigy Central Research, which is based in Trafford Park, Manchester, UK, provides an analytical microscopy service to other Ciba-Geigy companies.

One of the most significant problems that Central Research has to face is that the other Ciba-Geigy sites are scattered throughout the UK, so travel to and from Central Research in order to discuss the complex, high resolution images produced is a regular occurrence. One of the most frequent journeys, until recently, was between Central Research and Ciba-Geigy Bonded Structures in Duxford, which is near Cambridge. It seemed that the only way in which Central Research could improve its response was to move Cambridge nearer to Manchester, thereby effectively reducing the travel time.

With the introduction of the Photophone, the high-resolution images produced by the electron microscope are transmitted via an ordinary telephone line to Duxford. Conversations on the same telephone line can relay additional information quickly and easily.

With Photophone, the group had nothing to install, no computer language and no software to learn. Within 15 s the image at Central Research is in front of a colleague at Duxford without any loss of picture quality. When the analysis is complete, images can be stored for reviewing later, and can also provide a permanent record of images sent or received.

The Photophone – Picture Telephone is distributed by Cameron Communications Ltd and available around the world.

*For further information contact Elizabeth Stockwood, Cameron Communications Ltd, Communicate House, 50 Suttons Park Avenue, Reading, Berkshire RG6 1AZ, UK. Tel.: 0734 664611; fax: 0734 67716.*

### Synchron

Brochures from Beckman introduce the Synchron family of clinical analysers.

The first leaflet lists the features of the Synchron CX3, CX4 and CX5 analysers. The CX3 offers unparalleled throughput with STAT response to eight most commonly requested critical assessment tests, while the CX4 has been designed to complement such STAT systems as the CX3 and the Synchron AS. The stand-alone CX5 is able to deal with 28 chemistries, including metabolytes, electrolytes, proteins and drugs.

The second brochure describes the Beckman RPM System (results per minute) using Synchron CX3 inter-

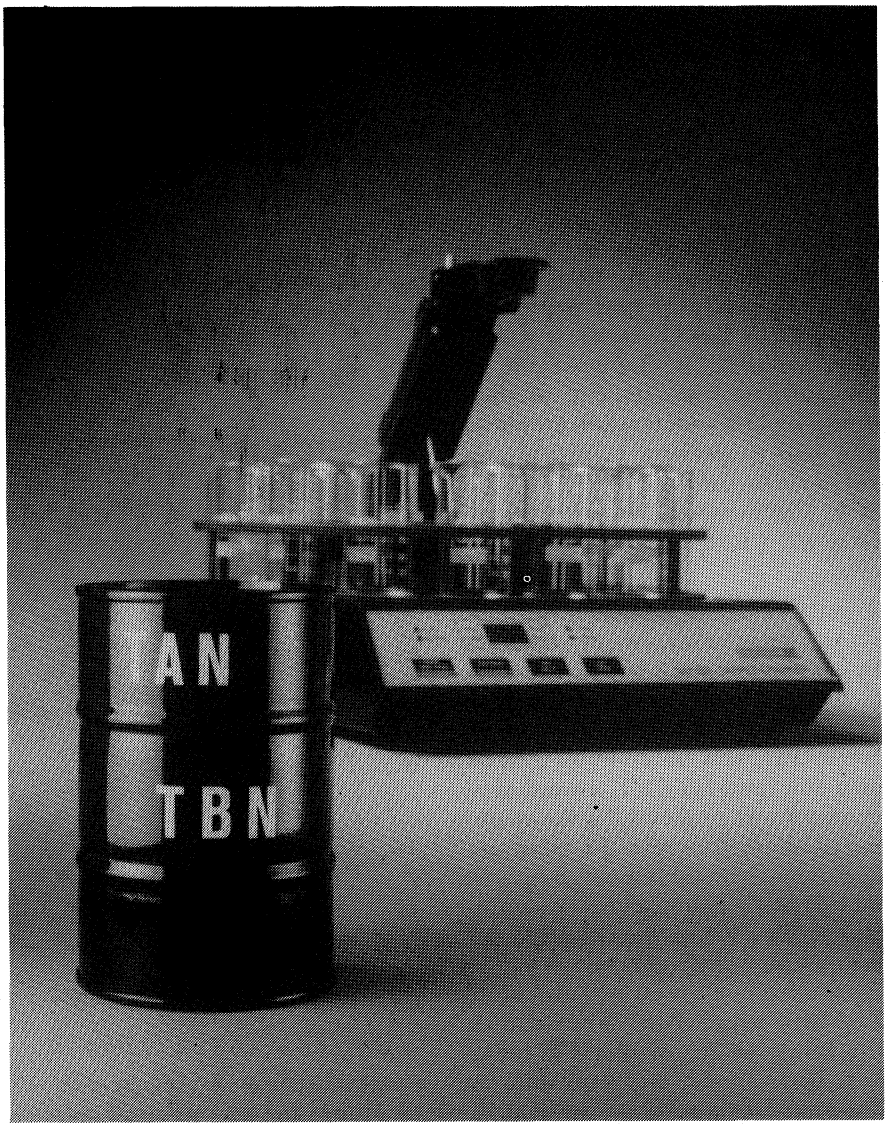

*For determining Total Acid Number (TAN) and Total Base Number (TBN) according to ASTM D 664, Radiometer Analytical offers the VIA102 TAN/TBN method module for installation in the TitraLab High Performance Titration Laboratory.*

*With the TAN/TBN method module, the TitraLab system is capable of calculating titration results from inflection points and fixed potentials found during a titration. Results are automatically calculated in mgKOH/g. For full information contact Radiometer Analytical A/S, 49 Krogshøjvej, DK 2880 Bagsvaerd, Denmark. Tel.: 01 69 63 11.*

faced with an existing random access analyser in the laboratory – a means of streamlining high volume testing and improving overall performance without the need to replace less effective equipment.

*Copies from Beckman, Progress Road, Sands Industrial Estate, High Wycombe, Buckinghamshire, UK. Tel.: 0494 41181.*

### Laboratory design brochure

Gallenkamp Contracts offer a comprehensive guide to the in range of services. Specializing in laboratory design and installations, the guide gives details about a specialist sales force, design team (using computer graphics), manufacture and installation services. Gallenkamp can complete a lab. with furniture and equipment on one order if required.

*The guide is available from Gallenkamp, Belton Road West, Loughborough, Leicestershire LE11 0TR, UK. Tel.: 0509 237371.*

### Sulphur dioxide analyser

Product literature is offered by Columbia Scientific Industries on its Model SA700 sulphur dioxide analyser. The continuous fluorescence sulphur dioxide analyser directly monitors $SO_2$ using a continuous UV source of intermediate intensity and high stability.

Its low-noise characteristics ensure rapid response while providing accurate and reliable data to better than 0·5% of full-scale, even on the most sensitive range.

*For a copy of the brochure or additional information, call Joe Herrmann on 800 531 5003 (outside Texas, USA) or 512 258 5191 (in Texas).*

### 'Education in Chemistry' on free circulation

From the March 1988 issue onwards, the Royal Society of Chemistry will be sending *Education in Chemistry*, its 25-year-old chemical education magazine, free of charge to every secondary school chemistry department in the UK.

*Education in Chemistry* is the only magazine in the UK aimed at teachers of chemistry at all levels, from 16-plus courses to the early stages of undergraduate education, with an emphasis on practical approaches and the sharing of ideas.

Although readership among secondary· teachers will increase, the bimonthly magazine will retain its present editorial policy and design. It will still be available on subscription (at £19·00 a year) to those who do not qualify for the free circulation or who wish to receive a personal copy, especially important to those in tertiary education and overseas.

### *The RSC*

As the professional body for chemistry in the UK, the Royal Society of Chemistry, which now has some 40 000 members, has Charter obligations to education. These include:

- Increasing public and government awareness of the central role of chemistry and chemists.
- Improving the image of chemistry.
- Improving chemical education and professional training.
- Ensuring that all young people have a balanced science curriculum with a proper emphasis on chemistry.
- Ensuring that an appropriate proportion of students are attracted to careers in chemistry.
- Encouraging a sufficient supply of science teachers qualified in chemistry.
- Campaigning for increased resources.

The RSC's ways of helping teachers include:

- *Education in Chemistry.*
- Schools Publications Service.
- Schools Liaison Officer and Schools Activities Committee.
- In-service training (INSET) through Saturday symposia and workshops for teachers; National conferences; Industry study tours.
- Curriculum development through Chemistry Plus; Salters' chemistry; National criteria.
- Resource materials – low cost publications; chemistry cassettes and computer data-bases (NERIS).
- Careers service.
- Chemistry olympiads and egg races.
- Active network of local sections.
- Funding of local education initiatives.
- SATROs, teachers centres and other school/industry link organizations.
- Promotion of chemistry through the media.

*Information about the RSC and free subscriptions to 'Education in Chemistry' from the Royal Society of Chemistry, Burlington House, Piccadilly, London W1V 0BN; paid subscription orders for 'Education in Chemistry' to the RSC Distribution Centre, Blackhorse Road, Letchworth, Hertfordshire SG6 1HN, UK.*

### Near infra-red analyser

Pacific Scientific have launched the Compscan 7000S near infrared standalone analyser in Europe. Previously available only in the USA, the system is designed to provide accurate quantative analysis on up to 100 products, and the company foresee many applications in laboratories and for process control.

The Compscan is simple to use; the operator selects the product and up to six calibrations are then displayed simultaneously on a large liquid crystal display. Once the sample cell is loaded into a sample transport module, results are displayed in seconds.

Results can be obtained as hard copy through an external line printer or data can be transmitted to an IBM PC or a compatible machine using NSAS-PC software.

Software is available for complete statistical spectral data analysis in real time. Control charts process monitoring can also be displayed. The NSAS-PC package also provides data storage and retrieval facilities for summary analysis.

A multiplex of up to six units at different locations, controlled through one PC, is possible. Calibration equation transfer is achieved by direct line and telephone modem, in addition to result monitoring.

A variety of sampling cells is available: these include a high fat/high moisture, slurry/paste cell. Also available are large granular cells, textile cells, reflectance cells and cuvettes.

*Full technical data from Pacific Scientific, 4 First Avenue, Marlow, Buckinghamshire, UK. Tel.: 06284 74074.*

### LIMS 2000 now on DEC VAX

LIMS (Laboratory Information Management Systems) 2000VX, an enhanced version of LIMS 2000, which Perkin-Elmer have announced is compatible with Digital Equipment Corporation's VAX computer series, as well as Concurrent Computer Corporation's 3200 series of minicomputers. This means that possible hardware compatibility worries, about which a number of laboratory managers had been concerned, are eliminated. Links to other departments already using other VAX system software for manipulation of data associated with LIMS are also made easier.

Each laboratory is unique and so its associated needs and operations are varied. Perkin-Elmer can tailor software for customers. A tailored system means better acceptance of the software as the laboratory does not necessarily have to adapt or adjust its working practices to LIMS. Any manufacturer's instruments can be used with LIMS 2000VX to provide a totally automated laboratory.

The possibility of using Digital hardware in conjunction with Perkin-Elmer's LIMS software and expertise in implementation of these systems will, according to Alan Williams, Product Manager for Laboratory Automation at Perkin-Elmer, 'make it much easier for would-be purchasers to make their decisions, should a rigid corporate computer purchasing policy exist'.

Perkin-Elmer has enhanced the reporting facilities of LIMS 2000VX; and the system is entirely configurable, which means that the user can create customized menus and write his own programs.

*For further information contact Perkin-Elmer Ltd, Post Office Lane, Beaconsfield, Buckinghamshire HP9 1QA, UK. Tel.: 04946 6161.*

### New chair in analytical chemistry

An award of £125 000 (to be spread over five years) from Philips Scientific has enabled Strathclyde University to establish a professorship in analytical chemistry.

The endowment strengthens Strathclyde's long-standing links with the Cambridge-based company,

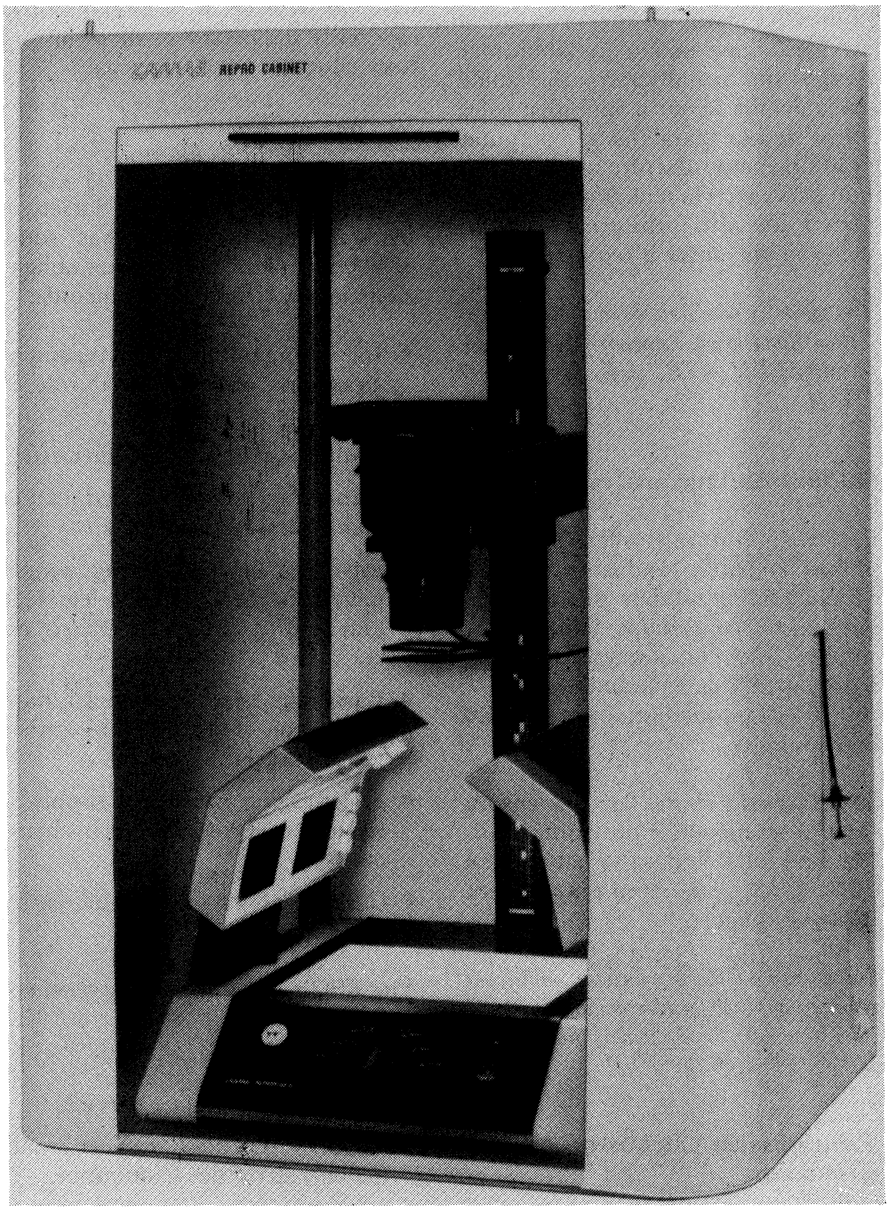

*CAMAG introduced the modular REPROSTAR II system in 1987 for photodocumentation in visible light, in short and long-wave UV light and, in the Transilluminator version, in mid-range UV light. A new camera is now available: the Mamiya RB 67 single lens reflex, which can be used with either Polaroid instant film or conventional film. Advantages of the Mamiya camera include easy focusing with mirror reflex system and a built-in automatic exposure meter. Details from Ch. Gfeller, CAMAG, Sonnenmattstrasse 11, CH-4132 Muttenz, Switzerland. Tel.: 061 61 34 34.*

which already supports basic research at the University through CASE awards, a fellowship, and through the loan and donation of scientific and analytical instrumentation.

The most recent result of that collaboration was the development by the University of the furnace autoprobe which forms a key part of one of Philips Analytical's latest instruments, the PU4900 series of atomic absorption spectrometers launched last year. The probe allows elemental analysis at greater levels of sensitivity than previously possible, and can cope with many complex sample matrices.

A recently published article highlighted a severe shortage of UK scientists in the field of analytical chemistry. It maintained that Britain, unlike other countries, has never really valued this field, that there are very few university chairs in analytical chemistry, and that it has always been seen 'as solving other people's problems when you haven't the wit to think of your own'. The role of the analytical chemistry is wide ranging, from the diagnosis of disease to the development of new products and processes for the general chemical industry, the microelectronics industry and the oil industry.

*The chair will be taken up in October 1988—details from Keith Andrews, Philips Analytical, Philips Scientific, York Street, Cambridge, CB1 2PX, UK. Tel.: 0223 358866; or Margaret Robertson, University of Strathclyde. Tel.: 041 552 4400, ext. 2182.*

### Oxides of nitrogen analyser

A brochure is now available from Columbia Scientific Industries, which outlines features, operational principles, design details, diagnostics and specifications of the model 1600 Mark III Oxides of Nitrogen Analyser.

A special section of the booklet explores comparisons between single and dual channel oxides of nitrogen analysers for ambient air measurements.

The 1600's reliability and performance is documented and the brochure shows how an innovative thermal converter design makes the 1600 Mark III one of the lowest cost NOx analyser to maintain.

The new thermal converter utilizes a replaceable catalyst cartridge. This low-cost cartridge can be easily replaced in less than 5 min; thus eliminating entire convertor replacement. CSI offers a choice of copper or molybdenum catalyst, each rated at over 2000 ppm-hours of operation.

*Details from Joe Herrmann. Tel.: 512 258 5191.*

### Extended capability for the Axxiom chromatography data systems

Quadrant Scientific Ltd have introduced a new interface for their Axxiom Chromatography Data Systems. With the new interface, both the Model 727 single-channel integrator and the Model 747 multichannel, multi-tasking data system can run on the IBM PS/2 series of computers.

The interface comprises a module with a special high-speed communications link to permit the real time acquisition and display of data from one to six detectors.

Simultaneous chromatogram editing and reprocessing are offered in the same way as on the XT and AT compatible computers.

*For further information contact Mark Wardle, Quadrant Scientific Ltd, Brunswick Road, Gloucester GL1 1JJ, UK. Tel.: 0452 504294.*

### Report: Oils and fats in the UK

Total UK consumption of yellow fats is in decline and fell to £685 M in 1986; consumers are tending to turn away from saturated products like butter and hard margarine in favour of polyunsaturated margarines and spreads that they perceive as 'healthier'. Butter's share of the market declined to 28% in 1986; margarines held 57% but spreads increased their share to almost 15%.

The cooking fats and oils market is fairly static in volume terms, reaching 181 500 tonnes in 1986. Consumption of vegetable oils is expanding at the expense of animal fats; liquid cooking oil dominates the sector with 42% and 41% by volume and value, respectively.

*Oils and Fats in the UK* is No. 27 in the Food Market Updates series by the Market Information Service at the Leatherhead Food RA. It covers the major sectors of the oils and fats market, giving consumption trends, market share and forecasts for future performance. The report costs £20 to members, £50 to non-members.

*Orders to The Leatherhead Food R.A., Randalls Road, Leatherhead, Surrey KT22 7RY, UK. Tel.: 0372 376761.*

### Autosampler

The Promis autosampler, a sample preparation station for HPLC analysers, is now available from Applied Chromatography Systems. The autosampler has the following features:

- Sample clean-up – this makes use of two extra six-way values and one six-position solvent selector to provide sample clean-up, preconcentration, or heart-cut back-flush configurations.
- Pre-column derivatization – the digital dispensing system gives accurate dilution and conditioning of samples.
- Injection modes – there are three injection modes, flushed loop, partial loop filling and micro litre pick up for negligible sample loss on very small sample volumes.
- Cooling bath – biological samples are often sensitive to temperature changes so a cryostatic bath can be used to keep the samples at 2–5°C.
- Satelite position – this can be used for needle flush, external standard, microlitre pick up, emergency samples and reagent pick-up.

*More information from Applied Chromatography Systems, The Arsenal, Heapy Street, Macclesfield, Cheshire SK11 7JB, UK. Tel.: 0625 34575; fax: 0625 616916.*

### Large-scale sample preparation

Analytichem International is introducing a new range of solid phase extraction columns for the rapid concentration and clean-up of trace compounds from very large or very dirty samples.

Available in 1 g/6 ml, 2 g/12 ml, 5 g/20 ml and 10 g/60 ml sorbent mass/column volume sizes, Mega Bond Elut high capacity/large volume columns eliminate the need for extra reservoirs when dealing with large volume samples.

Fully compatible with the Vac Elut SPS 24 sample processing station, the columns additionally provide the extra capacity necessary for complex or 'dirty' samples like food or soil.

Available in a wide range of phases including Florisil, the columns utilize Analytichem's Bondesil bonded silica sorbents – allowing direct method scale-up from standard Bond Elut extraction procedures.

*Further information from Richard Calverley, Analytichem International, P.O. Box 234, Cambridge CB2 1PE, UK. Tel.: 0223 328177.*

### GraphWare

Mettler's TA72 GraphWare software supports the processing of thermoanalytical measured values and curves as determined by the company's TA3000 System and prepared and transferred by the TC10A TA processor. The broad application area of this evaluation software covers not only the checking of materials and their development but also the investigation of thermal effects of new compounds. In all these investigations, theTA72 GraphWare offers the possibility to compare and simultaneously process several thermoanalytical curves.

All commands entered using the function keys are executed instantly by GraphWare on the colour monitor. TA Processor and computer can be operated independently; the system can thus be employed as a genuine multitasking station.

*Details from Mettler Instrumente AG, CH 8606 Greifensee, Switzerland.*

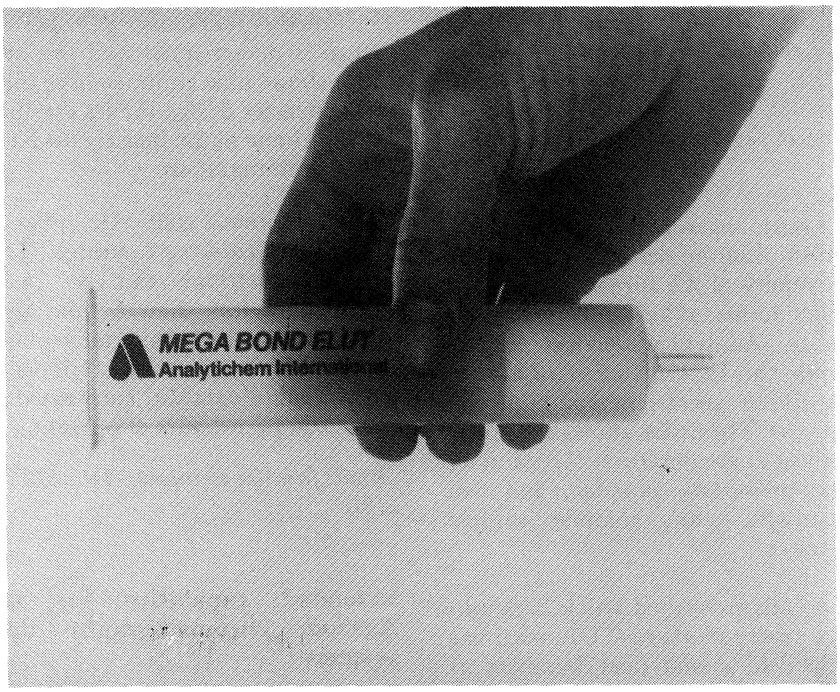

*Mega Bond Elut solid phase extraction columns from Analytichem International.*

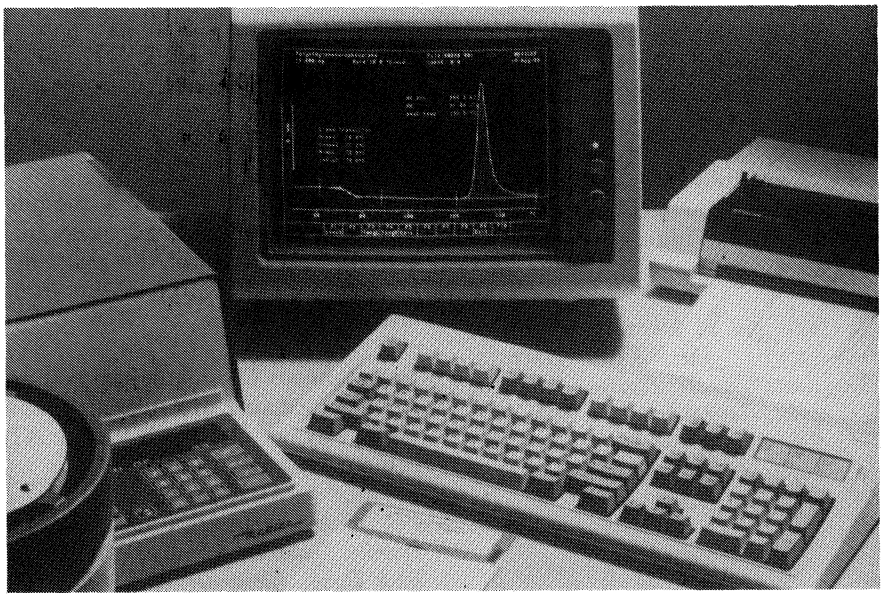

*Mettler TA72 GraphWare for comparing and processing thermoanalytical values and curves on a PC.*

### Queen's Award

A chemical's carrying specialist, Seawheel Ltd, has won a 1988 Queen's Award for Export Achievement.

Seawheel's award is based on its success in 'invisible exports' but it is noteworthy that in the last four years the Company's actual carryings to the Continent have doubled.

Seawheel attributed this growth to its successful marketing of a competitive service, supported by a policy of ploughing back profits into fleet investment, thereby ensuring that its container fleet capacity was able to keep pace with the growth in demand for its services.

Also, in the last three years Seawheel's overseas earnings have

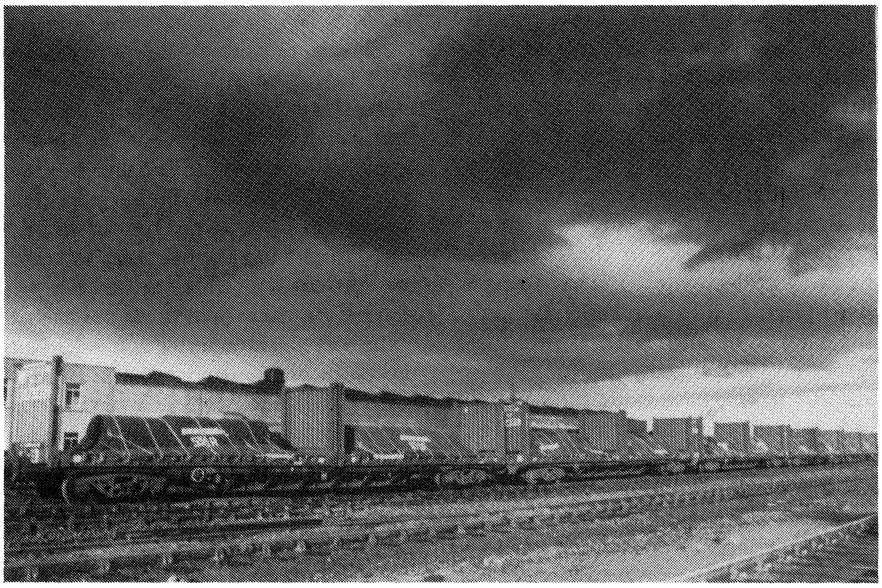

*These specially designed chemicals-carrying flatbed containers have contributed towards Seawheel Ltd winning a 1988 Queen's Award for Export Achievement. The unit load door-to-door container operator has expanded its business on the Continent by foreign earnings growth of 81% over the last three years.*

doubled, highlighting not only the Company's overall growth but also its effective marketing abroad.

An important factor in the company's recent growth has been the development and building up of its fleet of specialized containers and ISO flats. The Company's R&D programme has produced many design and construction firsts and this innovative approach has won them significant market shares, both in the general cargo sector and in such specialized fields as the carriage of chemicals and other deadweight traffics to and from Europe.

Seawheel's fleet of specialized ISO carriers is the largest in Europe, the company having been at the forefront in design and construction of these units for over two decades. Last year its 750 20 ft and 30 ft modules were complemented by the addition of a 300-strong fleet of 40 ft CargoFlats whose patented features enable them to be shipped either as 'drive through' trailers or as 'lift on/lift off' containers, transportable by road, rail or barge.

Seawheel's development of effective 'high and wide' drybox containers has been equally significant, in offering shippers new options for the economical transport of trailer-type or high-cube cargoes.

*Further information from Martin Isherwood, Imperial Studios, Imperial Road, London SW6 2AG.*

### Research grade FT-IR spectrometers

The 1700-X series offers an optional flexible external beam facility to optimize performance and sampling versatility for specialized interfaces and remote detector experiments. Based upon a new version of the 1700 Michelson interferometer, the advanced electro-optical design has produced a highly stable instrument with excellent signal-to-noise levels over the entire frequency range of 7200 to 370 $cm^{-1}$. Extended frequency range coverage (15800–30 $cm^{-1}$) is provided through a choice of optimized source, beamsplitter and detector configurations. Resolution on the Model 1760-X is variable from 0.5 to 64 $cm^{-1}$ and on the 1720-X from 2 to 64 $cm^{-1}$.

Two detector positions allow both the standard DTGS and a cooled detector to be mounted permanently in both internal and external sampling benches. Switching between detectors is software controlled. A photoacoustic detector is also available, and to minimize measurement time for each detector, the analyst has a choice of five scan speeds.

Full instrument control and data handling are provided by the Spectroscopy Terminal (1720-X) or by the Perkin-Elmer Model 7700 Professional Computer (1760-X) with FT-IR software. With the 1760-X, complete analyses can be fully automated with the OBEY macro programming language. The Model 7700 can control up to 16 optical units simultaneously. In addition, Model 1720-X Spectroscopy terminals can be added to create a true multiuser/multitasking FT-IR Research system or Multisystem.

*For further information, contact Perkin-Elmer Ltd, Post Office Lane, Beaconsfield, Buckinghamshire HP9 1QA, UK.*

### Reaction calorimeter expansion

With the new MP10 Medium Pressure Glass Reactor, the Mettler RC1 Reaction Calorimeter has been provided with an important accessory which offers expanded application possibilities.

The RC1 Reaction Calorimeter is a computer-controlled laboratory reactor for the running and development of chemical processes and their optimization with regard to safety and profitability under simulated plant conditions.

With the MP10 Medium Pressure Glass Reactor, such processes can now also be run in the pressure range vacuum to 10 bar over-pressure, thereby opening up an even broader

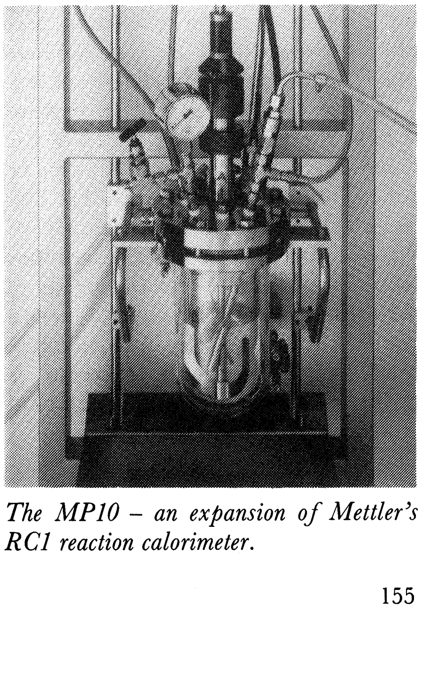

*The MP10 – an expansion of Mettler's RC1 reaction calorimeter.*

application spectrum for the user of the Mettler RC1 system.

Typical applications under pressure are experiments with reactive gases (for example hydrogenations, chlorinations or oxidations), as well as reactions in the presence of a protective or inert gas. Furthermore, the heat of vaporization for caloric evaluations can be suppressed – boiling or refluxing are prevented by increasing the total pressure.

The double-walled glass reactor is equipped with a thermostatable metal cover and a gas-tight magnetic clutch for the stirrer. In the temperature range −20 to 200 °C, isothermal, adiabatic or dynamic operations can be performed with an effective volume of 0·25 to 1 litre.

The MP10 is surrounded by a protective screen, which acts as a splinter guard and thus assures safety during work up to 5 bar over-pressure in the lab. In the pressure range 5 to 10 bar, safety reasons dictate the use of an autoclave room. A frame for the reactor and the stirrer motor is available for this purpose and allows operation from the control room.

*Details from Mettler Instrumente AG, CH 8606 Greifensee, Switzerland.*

### BSc course in computer-aided chemistry

A new BSc course in computer-aided chemistry at the University of Surrey, Guildford, UK, has received major additional financial and technological support in a funding programme sponsored by Glaxo Pharmaceuticals and Hewlett-Packard. The course, designed and implemented by the then Acting Head of the Chemistry Department, Dr John R. Jones, in conjunction with his colleague Dr S. Gabriel Buist and David Povey, aims to produce graduates whose training reflects the needs of the chemical industry today, where a considerable and growing involvement with computer systems is changing the face of the analytical laboratory.

The comparatively poor representation of analytical chemistry in UK tertiary education, with only five university and polytechnic schools all concentrating on higher degrees, has resulted in the demand of industry for well-trained analysts, especially at first degree level, far exceeding supply. Both Glaxo in the chemical industry and Hewlett-Packard as an instrument manufacturer have felt this shortage, and both companies agree on the need for long-term support to increase both the quality and quantity of graduates trained with the requirements of the chemical industry in mind.

The Chemistry Department at the University of Surrey designed their degree course in computer-aided chemistry in order to meet this need. The department's aim has been to provide the training and skills required by industry, and produce well-qualified graduates whose own employment prospects are excellent.

The next generation of computer tehnology will, in the words of course adviser Dr Vinter of Smith Kline & French 'be derived from the scientists versed in computers and not from the computer expert who takes his guidance from the scientist'. So tomorrow's analytical chemist must become skilled in computer programming, the interfacing of instruments and the application of data-base retrieval and management and computer graphics in order to develop and maximize the capability of the analytical instrumentation he or she will use.

In attaining these skills, however, it is essential not to allow the chemistry content of the course to be diluted. The new degree places particular emphasis on the hands-on approach, and includes a year working in industry. The Glaxo–HP support programme is primarily directed towards the analytical aspects of chemistry.

The hardware provided by Hewlett-Packard to the department includes an HP 8452A diode array UV/Visible spectrophotometer system together with a ChemStation and various accessories and peripherals. The range of software required to make the system operational has also been supplied by HP. With computational chemistry, molecular modelling, instrument interfacing and data analysis at the heart of the course, the department felt it necessary for each student to be able to access the system via their own personal microcomputer, Hewlett-Packard has therefore also supported the department with six Vectra worksations, configured into a local area network (LAN), bringing the total value of the hardware and software supplied to about £100 000.

The cost of installation and maintenance (over a five-year period) of the analytical hardware will be borne by Glaxo, who have pioneered the application of linear diode array spectrophotometry. Glaxo's support of the degree will include the transference of this application expertise to students and course tutors, ensuring that the syllabus and teaching remain abreast of developments and relevant to the needs of industry. This liaison programme will complement HP's support, and will provide for the Department's staff to receive full training in the principles and operation of the equipment.

The support and liaison programme will, according to Ken Leiper, Manager of Glaxo's Central Analytical Services, 'ensure that course tutors are kept aware of industry's changing requirement for high quality analytical information, and of how this need may be met effectively by the novel use of computer aided analysis systems'. David Aslin, General Manager of HP's Analytical Group in the UK considers that 'Both the specific application of linear diode array spectrophotometry and the enormous potential of local area networks are underexploited in UK industry. The use of LANs is increasing, and should increase, in industrial laboratories, and the University's commitment to training the skilled personnel who will put such systems into operation is imaginative and well deserving of our support'.

